# Draft Genome Sequences of Three Strains of *Geobacillus stearothermophilus* Isolated from a Milk Powder Manufacturing Plant

**DOI:** 10.1128/genomeA.00939-15

**Published:** 2015-10-15

**Authors:** Sara A. Burgess, Murray P. Cox, Steve H. Flint, Denise Lindsay, Patrick J. Biggs

**Affiliations:** a School of Food and Nutrition, Massey University, Palmerston North, New Zealand; b Statistics and Bioinformatics Group, Institute of Fundamental Sciences, Massey University, Palmerston North, New Zealand; c Fonterra Research and Development Centre, Palmerston North, New Zealand; d Infectious Disease Research Centre, Institute of Veterinary, Animal and Biomedical Sciences, Massey University, Palmerston North, New Zealand

## Abstract

Three strains of *Geobacillus stearothermophilus* (designated A1, P3, and D1) were isolated from a New Zealand milk powder manufacturing plant. Here, we describe their draft genome sequences. This information provided the first genomic insights into the nature of *G. stearothermophilus* strains present in the milk powder manufacturing environment.

## GENOME ANNOUNCEMENT

Thermophilic bacilli, such as *Geobacillus stearothermophilus*, are the most common bacteria that contaminate milk powder (1–3). Three strains of *G. stearothermophilus* (A1, P3, and D1) were isolated from the stainless steel surface of different sections of an evaporator in a milk powder manufacturing plant in New Zealand and were shown to vary in their ability to form biofilms and produce spores (1). The same study demonstrated that these strains can be differentiated by other physiological characteristics, such as their metabolism (by biochemical assays) and fatty acid profile and protein composition (e.g., by matrix-assisted laser desorption ionization–time of flight mass spectrometry). Genome sequences for thermophilic isolates originating from dairy manufacturing plants are available only for *Anoxybacillus flavithermus* (GenBank accession no. AMCM01) and *Geobacillus thermoglucosidasius* (GenBank accession no. AJJN01). For *G. stearothermophilus*, genome sequences are available only for non-dairy-associated strains (GenBank accession numbers JQCS01, JPYV01, and JALS01). Genome sequences for dairy-associated strains of *G. stearothermophilus* are needed to provide information on how these isolates have adapted to grow in the specific environment of milk powder manufacturing plants.

We therefore sequenced *G. stearothermophilus* strains A1, P3, and D1. Next-generation Illumina sequencing (250-bp paired-end reads) was performed on an Illumina MiSeq sequencer by New Zealand Genomics Ltd. (Massey University, Palmerston North, New Zealand). The number of reads generated for each strain were 3,749,346 for A1, 3,517,967 for P3, and 3,841,631 for D1. The reads were assembled into contigs separately for each strain using the Velvet assembler (version 1.2.10) (4). Strain A1 had 328 contigs >500 bp, with an estimated genome size of 3,012,236 bp; strain P3 had 328 contigs >500 bp, with an estimated genome size of 3,022,732 bp; and D1 had 295 contigs >500 bp, with an estimated genome size of 2,971,009 bp. The G+C content was 52.0% for A1, 52.0% for P3, and 52.2% for D1. Genome annotation was undertaken using Prokka (version 1.10) (5). We found 3,169 protein-coding sequences for A1, 3,200 for P3, and 3,171 for D1. Strains A1 and P3 contained 93 tRNA genes, whereas D1 had only 89. Initial analyses have shown that although all three strains belong to the same species, there were noticeable differences between them, particularly with D1 versus the other two strains, A1 and P3.

Further analyses of these genomes are ongoing to determine the different ways in which these strains have adapted to their dairy manufacturing environment.

### Nucleotide sequence accession numbers.

This whole-genome shotgun project has been deposited in GenBank under accession numbers LDNS01000001 (*G. stearothermophilus* A1), LDNT01000001 (*G. stearothermophilus* P3), and LDNU01000001 (*G. stearothermophilus* D1). The versions described in this paper are the first versions.
